# Assessing Eye Clinic Accessibility: A Study Validating and Applying the SiteWise Survey

**DOI:** 10.1167/tvst.13.10.37

**Published:** 2024-10-29

**Authors:** Jason Dossantos, Anne T. Riddering, Laura C. M. Ndjonko, Hassaam S. Choudhry, Nicolas Gasquet, Xiangrong Kong, Pradeep Y. Ramulu, Mona A. Kaleem

**Affiliations:** 1 School of Medicine and Health Sciences, George Washington University, Washington, DC, USA; 2 Henry Ford Health, Department of Ophthalmology, Detroit, MI, USA; 3 Department of Biological Sciences, Northwestern University, Evanston, IL, USA; 4 Rutgers New Jersey Medical School, Newark, NJ, USA; 5 Wilmer Eye institute, Johns Hopkins School of Medicine, Baltimore, MD, USA

**Keywords:** sitewise survey, low vision design, visual impairment, accessibility assessment, eye clinic accessibility

## Abstract

**Purpose:**

To validate the SiteWise survey as a reliable tool for assessing the accessibility of outpatient ophthalmology clinics for visually impaired patients and to compare accessibility between hospital-based and satellite clinics.

**Methods:**

This quality improvement study, conducted from January to December 2023, used the SiteWise survey to assess design features in seven satellite and two hospital-based clinics within the Wilmer Eye Institute network. Independent surveyors evaluated elements such as parking, sidewalks, entrances, and interior areas. Reliability was measured using Krippendorf's alpha, and accessibility scores were compared using generalized estimated equations, analyses of variance, and *t* tests.

**Results:**

The SiteWise survey demonstrated high reliability with a Krippendorf's alpha of 0.99. Hospital-based clinics had higher accessibility scores (mean 78.9%) compared to satellite clinics (mean 71.3%, *P* < 0.05). Areas such as hallways (mean 89%) and waiting areas (mean 87%) scored highest, whereas parking lots/sidewalks (mean 61%) and stairways (mean 61%) scored lowest, indicating significant room for improvement in these areas.

**Conclusions:**

The SiteWise tool is reliable and effective in identifying accessibility deficiencies in outpatient ophthalmology clinics. Although indoor areas generally scored well, outdoor and transitional spaces require significant enhancements to improve accessibility for visually impaired patients.

**Translational Relevance:**

This study bridges the gap between basic research and clinical care by providing a validated tool to assess and improve the accessibility of eye care facilities, ensuring they meet the needs of visually impaired patients.

## Introduction

As the prevalence of visual impairment increases globally,^1^^,^^2^ with an expected surge in the elderly population,^3^ the imperative for adaptive healthcare environments is more pronounced than ever. Particularly in the United States, where the number of individuals with significant visual impairment is projected to double by 2050,^4^ the challenge extends beyond mere acknowledgment to active accommodation. Accessibility refers to designing environments and services to be usable by all people to the greatest extent possible without the need for adaptation, while universal design is a broader approach that aims to improve human performance, wellness, and social participation by creating inclusive spaces that are accessible to people of all ability levels.^5^ This is crucial because older adults with visual impairment face increased risks of functional decline, necessitating comprehensive support that includes enhanced lighting, contrast, and accessibility in healthcare settings to maintain their independence and quality of life.^6^^,^^7^

Despite significant legislative strides, including the Architectural Barriers Act of 1968,^8^ the Americans with Disabilities Act of 1990 (ADA),^9^ and the United States signing the International Convention on the Rights of Persons with Disabilities in 2009,^10^ our healthcare infrastructure often falls short in addressing the nuanced needs of those with low vision. Critical elements such as optimal lighting, clear signage, and accessible information formats remain inadequately addressed, posing daily challenges for this population.^11^^–^^13^ Inaccessible healthcare facilities can result in missed appointments, delayed treatments, and increased anxiety and stress for visually impaired individuals, ultimately compromising their health outcomes.^14^^,^^15^ Recognizing this, the Henry Ford Health system pioneered a comprehensive survey in 2010, probing the efficacy of community facilities in supporting the elderly and visually impaired.^16^^,^^17^ This initiative not only spotlighted frequent key design deficiencies, but also improved the accessibility of their medical center, leading to the birth of the SiteWise low vision accessibility survey—a concerted effort to bridge the gap between legislative intent and practical, inclusive environments.^16^^,^^17^ Unlike the ADA Center's comprehensive but less specific accessibility checklists,^18^ the SiteWise Checklist offers a more detailed, vision-focused approach with specific guidelines and scoring systems to achieve higher standards of accessibility for individuals with low vision or blindness.

Our study seeks to validate the SiteWise survey through the analysis of inter-surveyor reliability, scrutinize the design features of a leading academic eye center's clinics, and increase awareness regarding the specific needs of patients with visual impairment and the elderly. By demonstrating the effectiveness of the SiteWise survey and providing this vision-focused tool, we advocate for inclusive and responsive designs that elevate the patient experience and improve accessibility standards in healthcare facilities.

## Methods

### Development of SiteWise

This low vision accessibility tool was developed with feedback from patients attending Henry Ford Health's Center for Vision Rehabilitation, who reported that after training they were more independent performing their activities of daily living at home but often complained of continued difficulty in the community. Difficult tasks included reading in darker restaurants, churches, and libraries, navigating obstacles such as curbs, freestanding signs, and furniture in libraries, community centers, and churches, and writing at the bank. A pilot program to assess low-vision accessibility in the community was developed, and the survey was tested on ten sites in the metro-Detroit area. It was well received by business owners and community leaders, and, with a grant from the Community Foundation for Southeast Michigan,^19^ the project was expanded to survey 30 sites in three counties in the metro-Detroit area. Once the survey was complete, each site was provided with recommendations to improve their facilities for seniors and individuals with visual impairments.

The original SiteWise accessibility tool was created in 2010 and updated for clarity in 2022. The survey consists of 83 graded items, evaluating eight sites within the outpatient clinical setting: (1) parking lots/sidewalks, (2) entrances/exits, (3) hallways, (4) stairways, (5) waiting areas, (6) customer service areas, (7) restrooms, and (8) examination rooms. Each item can be graded as “Yes,” “No,” or “Not Applicable.” If the question does not apply to the building or room (e.g., no stairs, no elevator, no windows), “Not Applicable” is checked. Supplementary Figure S1 displays an example of the parking lot/sidewalk assessment (first page) and the scoring checklist with the SiteWise standard threshold for outstanding (gold standard; 90% and higher), adequate (silver standard; 71%–89%), and minimum (bronze standard; 60%–70%) accessibility (last page). The full survey can be found in Supplementary Figure S1.

### Facility Evaluation Methods

The Wilmer Eye Institute's seven satellite clinics and two hospital-based clinics were evaluated using the SiteWise checklist. During standard business hours, independent surveyors assessed each facility. Because of the presence of multiple entrances, rooms, and objects at each facility, surveyors predetermined the specific areas to assess before conducting individual assessments. To ensure the selected routes were representative of typical patient experiences, preliminary observations and consultations with facility staff, including ophthalmic technicians and administrative personnel, were conducted to identify the most frequently used paths by patients. These routes included paths from parking areas to main entrances, primary corridors leading to waiting rooms and examination areas, and other high-traffic zones commonly traversed by patients.

In conducting these assessments, several key elements were measured. “Contrast” was defined as the differential in color, brightness, or texture between an element (e.g., sign text) and its backdrop, influencing its readability. Graders assessed contrast using examples from the training material (Supplementary Fig. S2). Light intensity in lux was gauged using a digital light meter (Dr.meter LX1330B; Dr.meter, Newark, CA, USA). Additionally, measurements of size and distance were approximated to the nearest half-inch.

### Surveyor Training and Proficiency Evaluation

Two graders without a background in eye care or medicine were trained in the survey methods. This hour-long training ensured both graders grasped the evaluation procedure (Supplementary Fig. S2). To validate their comprehension, the graders independently evaluated a satellite clinic, later comparing findings to establish consistency. This preliminary evaluation was excluded from the study. Throughout the main data collection from nine facilities, graders conducted assessments independently without mutual discussion of results.

### Statistical Analyses

To evaluate inter-grader agreement, Krippendorff's alpha (KA) values,^20^ with 95% confidence intervals, were determined across various units: the total sample, clinic type (satellites vs. hospitals), item category (e.g., presence of hospital components; print size, font, boldness; contrast and glare), individual clinics, and specific sites. Scores for each site and clinic were calculated as the proportion of “Yes” responses to the total number of questions answered as “Yes” or “No,” excluding questions that were not applicable. Subsequently, an average score from both judges was computed. Using Generalized Estimated Equations, these proportions were compared between satellites and hospitals, accounting for repeated measures at sites. Differences in mean scores across individual satellite clinics, within the entire sample, and separately within satellites and hospitals were analyzed using analysis of variance (ANOVA). A *t*-test assessed score variations between individual hospital clinics. All tests were two-tailed with a significance level set at *P* < 0.05. Analyses were performed in SAS 9.4.

## Results

Our study encompassed a diverse array of healthcare facilities, each representing a unique microenvironment within the broader context of visual impairment accessibility challenges. Specifically, the evaluation included seven satellite clinics and two hospital-based clinics within the Wilmer Eye Institute network, each varying in design, patient population, and service offerings. These facilities were carefully selected to provide a comprehensive overview of current accessibility standards and practices, reflecting the real-world experiences of visually impaired patients navigating these spaces.

Intergrader agreement was examined across all evaluated facilities, yielding a KA value of 0.99, indicative of almost perfect consensus (Table 1).^21^ This high level of consistency was mirrored in the specific facility evaluations, with agreement values ranging from 0.97 to 1.00. The uniformity in scoring extended to the site and item levels as well, with agreement ranges of 0.96 to 1.00 observed in both categories (Tables 1 and 2, respectively).

**Table 1.** Agreement Between SiteWise Surveyors Within Facilities and Sites at Wilmer Eye Institute Facilities

	Questions	KA Value	95% CI
Facility Name			
Total sample		0.99	(0.97, 1.00)
Clinic types			
Satellites (n = 7)		0.99	(0.97, 1.00)
Hospitals (n = 2)		0.99	(0.96, 1.00)
Site name			
Customer service	All	0.98	(0.94, 1.00)
Entrances/exits	All	0.99	(0.96, 1.00)
Examination rooms	All	0.98	(0.94, 1.00)
Hallways	All	1.00	(1.00, 1.00)
Parking lots	All	0.98	(0.95, 1.00)
Restrooms	All	0.98	(0.93, 1.00)
Stairways	All	1.00	(1.00, 1.00)
Waiting areas	All	0.96	(0.88, 1.00)

CI, confidence interval.

**Table 2.** Agreement Between Surveyors Among Similar Items on SiteWise Survey

Agreement Within Similar Questions	Category Number	Questions	Sites	KA Value	95% CI
Total sample				0.99	(0.97, 1.00)
Question Categories					
Presence of hospital components	1	E1, E4, E12, EX8, P1, P9, R1, R8, R9, S5, W4, W5	Entrances/Exits, Examination Rooms, Parking Lots/Sidewalks, Restrooms, Stairways, Waiting Areas	1.00	(1.00, 1.00)
Print size, font, boldness	2	C12, C13, C14, C15	Customer Service	1.00	(1.00, 1.00)
Basic contrasting colors	3	E10, E2, P4, R10, S1, S2, W3	Entrances/Exists, Parking Lots/Sidewalks, Restrooms, Stairways, Waiting Areas	1.00	(1.00, 1.00)
High-contrast markings that aren't paint or print	4	C10, C9, E3, EX3, EX9, P8	Customer Service, Entrances/Exists, Examination Rooms, Parking Lots/Sidewalks	1.00	(1.00, 1.00)
Reduction of glare	5	C2, C8, E11, H6, S4, W7	Customer Service, Entrances/Exists, Hallways, Stairways, Waiting Areas	1.00	(1.00, 1.00)
Printed high contrast	6	C6, C17, E5, EX11, EX6, H3, R3, S6	Customer Service, Entrances/Exits, Examination Rooms, Hallways, Restrooms, Stairways	1.00	(1.00, 1.00)
Painted higher contrast	7	P2, P3, P5, P6, P7	Parking Lots/Sidewalks	0.96	(0.89, 1.00)
Eye-level reading	8	C5, EX2	Customer Service, Examination Room	1.00	(1.00, 1.00)
Permanent objects contrasting colors	9	C4, E8, EX5, H5, P11, R12, W2	Customer Service, Entrances/Exists, Examination Rooms, Hallways, Parking Lots/Sidewalks. Restrooms, Waiting Rooms	0.96	(0.89, 1.00)
Walkways	10	C3, E7, EX4, H4, P10, R11, W1	Customer Service, Entrances/Exits, Examination Rooms, Hallways, Parking Lots/Sidewalks, Restrooms, Waiting Areas	1.00	(1.00, 1.00)
Lighting criteria of SiteWise	11	C1, E9, H1, R4, R5, R6, R7, S3, W6	Customer Service Areas, Entrances/Exits, Hallways, Restrooms, Stairways, Waiting Areas	0.98	(0.93, 1.00)
Font and print size criteria of SiteWise	12	C11, C16, C7, E6, EX1, EX10, EX7, H2, R2, S7	Customer Service Areas, Entrances/Exits, Examination Rooms, Hallways, Restrooms, Stairways	0.96	(0.90, 1.00)

CI, confidence interval.

Hospitals recorded an average SiteWise survey score of 78.9%, marginally surpassing the satellite clinics, which averaged at 71.3% (Table 3). This distinction between hospitals and satellite clinics was statistically significant, with a mean difference of −7.7% (*P* = 0.005). Interestingly, the intra-group comparison of survey scores were not statistically significant. Based on the key for SiteWise standards, three of the seven clinics were classified as silver standard, whereas four were rated as bronze standard. Both hospitals were also classified as silver standard.

**Table 3.** Mean SiteWise Survey Score Among Wilmer Eye Institute Hospitals Versus Satellites

Parameter	Mean	SE	95% Confidence Limits	*Z*	*P* Value
Satellites	71.3%	2.0%	67.2%–75.3%		
Hospitals	78.9%	4.5%	69.4%–88.4%		
Mean Difference	−7.7%	2.7%	−13.0% to −2.3%	−2.8	0.0048^*^

SE, standard error.

* *P* value < 0.05 is statistically significant.

An in-depth analysis of mean site scores across all facilities revealed important distinctions (Table 4). Within the total sample, the ANOVA highlighted statistically significant differences in mean score across clinic sites (Table 4; *F*(7) = 7.5, *P* < 0.0001). The highest scores, with means of 89.3%, 87.1%, and 79.3%, were observed in “Hallways,” “Waiting Areas,” and “Customer Service Area,” respectively. Conversely, “Parking lot/sidewalks” and “Stairways” registered lower means at 60.9% and 61.1%, respectively. Within satellite clinics, the ANOVA highlighted significant mean site score differences (Table 5; *F*(7) = 6.3, *P* < .0001). Specific sites, like “Hallways” (mean = 86.1%) and “Waiting Areas” (mean = 83.4%), outperformed others, whereas “Parking lot/sidewalks” and “Stairways” scored 56.2% and 61%, respectively. Hospital site scores mirrored these discrepancies (Table 5).

**Table 4.** Mean Site Score of Wilmer Eye Institute Facilities Within Overall SiteWise Survey Sample

Source	Mean	Std Dev	DF	Anova SS	Mean Square	*F* Value	*P* Value
Outpatient clinic			7	0.8	0.1	7.5	<0.0001^*^
Clinic name							
Customer service area	79.3%	4.3%					
Entrances/exits	63.5%	8.8%					
Examination rooms	67.9%	8.0%					
Hallways	89.3%	14.3%					
Parking lot/sidewalks	60.9%	16.7%					
Restrooms	74.6%	11.4%					
Stairways	61.1%	17.4%					
Waiting areas	87.1%	13.0%					

DF, degrees of freedom; SS, sum of squares.

* *P* value <0.05 is statistically significant

**Table 5.** Differences in Mean Site Scores Within Wilmer Eye Institute Hospitals and Satellite Clinics

	Mean and SD						
Source	Hospital	Satellite	DF	ANOVA SS	Mean Square	*F* Value	*P* Value
Hospitals			7	0.3	0.04	2.1	0.2
Satellite clinic			7	0.6	0.09	6.3	**<0.0001** ^*^
Site							
Customer service area	85.3% ± 4.2%	77.6% ± 2.5%					
Entrances/exits	67.7% ± 0.0%	62.6% ± 10.0%					
Examination rooms	72.7% ± 0.0%	66.5% ± 8.7%					
Hallways	100% ± 0.0%	86.2% ± 15.0%					
Parking lot/sidewalks	77.1% ± 20.6%	56.2% ± 13.8%					
Restrooms	67.8% ± 17.3%	76.6% ± 10.2%					
Stairways	61.7% ± 30.6%	61.0% ± 15.7%					
Waiting areas	100% ± 0.0%	83.4% ± 12.4%					

DF, degrees of freedom; SS, sum of squares.

* *P* value <0.05 is statistically significant.

Bolded *P*-values represent statistically significant values.

## Discussion

In our assessment, we used the SiteWise tool, a novel instrument specifically developed to evaluate low vision accessibility in outpatient medical clinics. Several previous tools and studies have delved into factors crucial for enhancing access for low vision patients^18^^,^^22^^–^^29^; the SiteWise tool stands out as the first instrument tailored to gauge the accessibility of clinics for visually impaired individuals. Notably, its design emphasizes elements such as lighting and contrast, which could potentially create challenges for this demographic. Intriguingly, these aspects may also be problematic for those without any visual impairment. A standout feature of the SiteWise tool is that it was developed through direct feedback from low-vision patients. Each criterion reflects real-world challenges encountered by patients within a clinical setting, highlighting that trained visual skills and prescribed optical aids are insufficient if the environment remains inaccessible. This approach aligns with the proposed ICF standards by Billiet et al.,^30^ which stress the significance of considering both environmental and personal factors when aiming to create an inclusive healthcare environment for individuals with vision loss. Moreover, the survey's design facilitates easy administration by medical personnel such as clinic managers or facilities staff—a crucial feature because low vision services may not always be provided by ophthalmologists.^25^

### Validation of the SiteWise Survey

The applicability of this instrument to our sample and the high interobserver reliability demonstrates the instrument's validity. We observed almost perfect agreement (0.96 to 0.99)^21^ between two non-expert graders in all facilities, sites, and items (Tables 1 and 2). This high level of agreement between graders may partially reflect the initial training (which included visual examples of contrast) and preliminary evaluation that was completed to validate their comprehension. Additionally, objectively graded items such as low light (measured by lux meter) and font size (measured in inches) may contribute to the level of consensus.

### Accessibility Evaluation in Ophthalmology Clinics

We observed a statistically significant difference in accessibility scores between hospitals and satellite clinics, with hospitals achieving a slightly higher average score of 78.9%, in contrast to satellite clinics which averaged 71.3% (Table 3). This disparity may be attributed to factors such as the age of the facilities, the design and renovation history, and the scale of services offered, which may affect adherence to contemporary ADA standards. Furthermore, the lower average score for satellite clinics could be influenced by the limited scope of renovations possible outside of and within multipurpose building suites. It is notable that the Johns Hopkins Hospital eye clinic, which includes a clinic specifically tailored for low-vision patients, attained a high score of 82.3%, thereby underscoring the potential impact of specialized design considerations. The observed discrepancy also may be influenced by the small sample size, with only two hospitals versus seven satellite clinics evaluated. Therefore, although all facilities are mandated to be accessible by the ADA, the degree to which they meet or exceed these standards can vary considerably.

In addition, our study identified significant variations in accessibility scores across different sites within all evaluated facilities. “Hallways,” “Waiting Areas,” “Customer Service Areas,” and “Restrooms” achieved the highest scores, indicating adequate accessibility (silver standard) by SiteWise Standards (Supplementary Fig. S1). On the other hand, “Parking lot/sidewalks,” “Stairways,” “Entrances/exits,” and “Exam Rooms” scored lower, receiving the lowest accessibility score (bronze standard). These results emphasize that although internal aspects of a facility like hallways and waiting areas may be adequately tailored for low vision accessibility, outdoor and transitional areas require attention the most. These observations also suggest that architectural and design considerations might often be inward-focused, with greater emphasis on internal environments rather than holistic facility design.

There was a variation in the item deficits for each low-scoring site. Entrances and exits often lacked sufficient contrast against metal door dividers, complicating the ability of visually impaired individuals to discern different areas, a challenge compounded for the elderly who are at a higher risk for fall-related ocular trauma.^31^ Stairways, frequently suffering from inadequate lighting below the recommended 400–600 lux, absence of contrasting railings, and non-contrasted step edges, not only impede visibility for the visually impaired but also significantly increase the risk of falls, which can result in serious ocular injuries. In fact, falls are the leading cause of injury-related emergency department visits and accidental deaths among older adults, with an estimated 3 million emergency department visits, over 950,000 hospitalizations, and approximately 32,000 deaths annually because of fall-related injuries.^32^ Parking areas and pathways often missed high-contrast curb edges and clear markings for inclines and declines, which are essential to prevent missteps and falls, whereas unobstructed walk paths are crucial for safe navigation. Examination rooms predominantly displayed undersized room numbers and lacked contrast on chairs, instrument stands, and tables, further contributing to the hazard landscape for visually impaired patients. Overall, these features have critical implications for safety and accessibility, particularly for the visually impaired and elderly.

These identified deficits provide actionable insights for facility managers and designers, emphasizing the need for individualized solutions rather than a one-size-fits-all approach. Each area of a facility may require unique modifications, informed by the feedback from those who navigate these spaces regularly—in this instance, the visually impaired patients. For example, the strategic application of contrasting colors and improved lighting could mitigate fall risks, especially during winter months when fall-related injuries peak.^33^ Furthermore, our sample revealed that four clinics were classified as bronze according to the SiteWise standard, guiding us to target these clinics for quality improvement. Ultimately, a concerted effort to redesign these spaces with the input of end-users can significantly reduce injury rates and improve overall safety for all patrons. These recommendations for facility-specific improvements, derived from the direct experiences of visually impaired individuals, paves the way for a more expansive conversation on how these principles can be generalized to enhance accessibility across all public and private spaces.

Although our research has concentrated on accessibility within eye clinics, the implications likely extend to a variety of public and private spaces frequented by visually impaired individuals. In line with the Inclusive Design framework as detailed by Gomez et al.,^34^ which prioritizes usability for people regardless of their visual abilities, our findings advocate for design modifications that span beyond healthcare settings. This framework entails creating environments where visual contrasts, tactile cues, auditory signals, and intuitive navigation are integral, thus accommodating the full spectrum of sensory engagement.^34^ Such enhancements are not exclusive to those with visual impairments; they benefit a broader population, including the elderly and patients with sensory deficits. Future research should thus examine the degree to which these inclusive design features are present or absent in a range of facilities, comparing these to the deficits we have documented in eye clinics. This would enable a more nuanced understanding of how the built environment can either support or hinder the independence and safety of visually impaired individuals, reinforcing the need for universally accessible design that aligns with the evolving demographics and diverse capabilities of the global population.

This study's limitations warrant careful consideration. Primarily, the evaluations were constrained to a single point in time, which may not capture the full spectrum of accessibility challenges. Moreover, our comparative analysis relied solely on non-specialist surveyors; a more comprehensive evaluation would benefit from including both specialists and non-specialists to provide a nuanced understanding of the tool's clinical relevance. Furthermore, the research used a convenience sample drawn from clinics within one healthcare system in a specific geographic area, which may limit the generalizability of our findings to other medical or hospital outpatient facilities, especially in varied locales. These considerations highlight the necessity for expanded research across a broader and more varied range of facilities to thoroughly validate the clinical applicability of our assessment tool.

## Conclusions

The SiteWise tool has revealed key areas for improvement in clinic accessibility for the visually impaired. Although indoor spaces like hallways and waiting rooms are generally accessible, outdoor, and transitional areas in our sample need attention. The disparities in scores between hospitals and satellite clinics further highlight the importance of tailored solutions for different facilities. These findings not only provide actionable insights for eye clinics but also underscore the broader need for inclusive design in various community spaces. Our application of this instrument to our clinics demonstrates its validity and its utility in quality improvement projects. Healthcare clinics that care for the visually impaired must advocate for more accessible design elements, regardless of the services provided. Future studies should broaden their scope, considering diverse facilities and broader participant involvement, to ensure universally accessible environments.

## Supplementary Material

Supplement 1

Supplement 2

## References

[1] Vision impairment and blindness. Available at: https://www.who.int/news-room/fact-sheets/detail/blindness-and-visual-impairment. Accessed May 26, 2023.

[2] GBD 2019 Blindness and Vision Impairment Collaborators, Vision Loss Expert Group of the Global Burden of Disease Study. Trends in prevalence of blindness and distance and near vision impairment over 30 years: an analysis for the Global Burden of Disease Study. *Lancet Glob Health*. 2021; 9(2): e130–e143. 33275950 10.1016/S2214-109X(20)30425-3 PMC7820390

[3] Congdon N, O'Colmain B, Klaver CCW, et al. Causes and prevalence of visual impairment among adults in the United States. *Arch Ophthalmol*. 2004; 122: 477–485. 15078664 10.1001/archopht.122.4.477

[4] Varma R, Vajaranant TS, Burkemper B, et al. Visual impairment and blindness in adults in the United States: demographic and geographic variations from 2015 to 2050. *JAMA Ophthalmol*. 2016; 134: 802–809. 27197072 10.1001/jamaophthalmol.2016.1284 PMC5116104

[5] Mosca EI, Steinfeld E, Capolongo S. Universal Design assessment tool to promote well-being and inclusion in healthcare environment. *Eur J Public Health*. 2020; 30(Suppl 5): ckaa165.589.

[6] Kaldenberg J, Smallfield S, Planche E, Heller S, Rothman E. Mapping the Gap: Understanding the Need for Occupational Therapy Among Older Adults With Visual Impairment. *Am J Occup Ther*. 2023; 77: 5. 10.5014/ajot.2023.050219 37774100

[7] Demmin DL, Silverstein SM. Visual impairment and mental health: unmet needs and treatment options. *Clin Ophthalmol*. 2020; 14: 4229–4251. 33299297 10.2147/OPTH.S258783 PMC7721280

[8] U.S. Access Board - Architectural Barriers Act. Available at: https://www.access-board.gov/law/aba.html. Accessed August 25, 2023.

[9] Americans with Disabilities Act of 1990, As Amended. ADA.gov. Available at: https://www.ada.gov/law-and-regs/ada/. Accessed August 25, 2023.

[10] Convention on the Rights of Persons with Disabilities. Available at: https://www.ohchr.org/en/instruments-mechanisms/instruments/convention-rights-persons-disabilities. OHCHR. Accessed July 20, 2024.

[11] Hoogsteen KMP, Szpiro S. A holistic understanding of challenges faced by people with low vision. *Res Dev Disabil*. 2023; 138: 104517. 37099881 10.1016/j.ridd.2023.104517

[12] Burton MJ, Ramke J, Marques AP, et al. The Lancet Global Health Commission on Global Eye Health: vision beyond 2020. *Lancet Global Health*. 2021; 9(4): e489–e551. 33607016 10.1016/S2214-109X(20)30488-5 PMC7966694

[13] Disability and Health Disability Barriers | CDC. Available at: https://www.cdc.gov/ncbddd/disabilityandhealth/disability-barriers.html. Accessed May 26, 2023.

[14] Upadhyay V, Balakrishnan M. Accessibility of healthcare facility for persons with visual disability. In: *2021 IEEE International Conference on Pervasive Computing and Communications Workshops and Other Affiliated Events (PerCom Workshops)*. 2021: 87–92.

[15] How Does Disability Affect Access to Health Care for Dual Eligible Beneficiaries? Available at: https://www.cms.gov/files/document/how-does-disability-affect-access-non-duals-dh.pdf. Accessed July 20, 2024.

[16] American Academy of Ophthalmology. Adapting your office environment for senior safety and comfort. Available at: https://www.aao.org/eyenet/academy-live/detail/adapting-your-office-environment-senior-safety. Accessed October 29, 2023.

[17] DBusiness Magazine. Henry Ford Vision Center Helps Facilities to be Senior-Friendly. Published March 21, 2011. Accessed October 29, 2023. https://www.dbusiness.com/people/henry-ford-vision-center-helps-facilities-to-be-senior-friendly/

[18] Northwest ADA Center. Accessibility Checklists. Available at: https://nwadacenter.org/toolkit/accessibility-checklists. Accessed July 20, 2024.

[19] Stallings B . Community foundation for Southeast Michigan makes $356,600 in grants. Available at: https://www.crainsdetroit.com/article/20080710/SUB/184945817/community-foundation-for-southeast-michigan-makes-356600-in-grants. Accessed October 30, 2023.

[20] Krippendorff K. Computing Krippendorff's Alpha-Reliability. Available at: https://www.semanticscholar.org/paper/Computing-Krippendorff%27s-Alpha-Reliability-Krippendorff/23f48132c4629b134c0d60fb668e645af99076fb. Accessed October 29, 2023.

[21] Landis JR, Koch GG. The measurement of observer agreement for categorical data. *Biometrics*. 1977; 33: 159–174. 843571

[22] Swenor BK, Yonge AV, Goldhammer V, Miller R, Gitlin LN, Ramulu P. Evaluation of the Home Environment Assessment for the Visually Impaired (HEAVI): an instrument designed to quantify fall-related hazards in the visually impaired. *BMC Geriatr*. 2016; 16: 214. 27938346 10.1186/s12877-016-0391-2 PMC5148906

[23] Guo X, Boland MV, Swenor BK, Goldstein JE. Low vision rehabilitation service utilization before and after implementation of a clinical decision support system in ophthalmology. *JAMA Network Open*. 2023; 6(2): e2254006. 36735257 10.1001/jamanetworkopen.2022.54006 PMC9898817

[24] Iezzoni LI, Rao SR, Ressalam J, Bolcic-Jankovic D, Campbell EG. Incidence of accommodations for patients with significant vision limitations in physicians’ offices in the US. *JAMA Ophthalmol*. 2022; 140: 79–84. 34854912 10.1001/jamaophthalmol.2021.5072 PMC8640945

[25] Jose J, Thomas J, Bhakat P, Krithica S. Awareness, knowledge, and barriers to low vision services among eye care practitioners. *Oman J Ophthalmol*. 2016; 9: 37–43. 27013827 10.4103/0974-620X.176099 PMC4785707

[26] Sarika G, Venugopal D, Sailaja MVS, Evangeline S, Krishna Kumar R. Barriers and enablers to low vision care services in a tertiary eye care hospital: a mixed method study. *Indian J Ophthalmol*. 2019; 67: 536–540. 30900589 10.4103/ijo.IJO_1215_18 PMC6446634

[27] Ejukonemu BOM, Akpalaba R. Determination of a standard continuous-text print size for people with low vision. Available at: https://www.semanticscholar.org/paper/Determination-of-a-standard-continuous-text-print-Ejukonemu-Akpalaba/f98be170f923b690b236ea698c7c3201eeae43e7. Accessed January 6, 2022.

[28] Rodrigues R, Coelho R, Tavares JMRS. Healthcare signage design: a review on recommendations for effective signing systems. *HERD*. 2019; 12(3): 45–65. 30501404 10.1177/1937586718814822

[29] O'Day BL, Killeen M, Iezzoni LI. Improving health care experiences of persons who are blind or have low vision: suggestions from focus groups. *Am J Med Qual*. 2004; 19(5): 193–200. 15532911 10.1177/106286060401900503

[30] Billiet L, van Nispen RMA, De Baets S, de Vries R, Van de Velde D, van der Aa HPA. The first step in developing an international classification of functioning, disability and health core set for vision loss: a systematic review. *Ophthalmic Physiol Opt*. 2024; 44: 413–425. 38251457 10.1111/opo.13269 PMC12872645

[31] Halawa O, Mitchell W, Zebardast N. Fall-related eye injury among older adults in the United States. *Am J Ophthalmol*. 2021; 229: 82–89. 33848534 10.1016/j.ajo.2021.03.063

[32] Moreland B, Kakara R, Henry A. trends in nonfatal falls and fall-related injuries among adults aged ≥65 years—United States, 2012–2018. *MMWR Morb Mortal Wkly Rep*. 2020; 69: 875–881. 32644982 10.15585/mmwr.mm6927a5 PMC7732363

[33] Choudhry HS, Zhu A, Shaikh S, Zaki H, Masket S, Law SK. Epidemiology of consumer-product-related ocular injuries in the geriatric population in the United States. *Ophthalmol Ther*. 2024; 13: 367–384. 37995015 10.1007/s40123-023-00852-4 PMC10776527

[34] Gomez J, Langdon P, Bichard JA, Clarkson P. Designing Accessible Workplaces for Visually Impaired People. In: Langdon PM, Lazar J, Heylighen A, Dong H, eds. *Inclusive Designing: Joining Usability, Accessibility, and Inclusion*. Berlin, Germany: Springer International Publishing. 2014: 269–279.

