# Supplementary material for: Assessing Eye Clinic Accessibility: A Study Validating and Applying the SiteWise Survey
Source: Transl Vis Sci Technol. 2024 Oct 29;13(10):37. doi: 10.1167/tvst.13.10.37 (PMC11534020; doi:10.1167/tvst.13.10.37)
Supplement: Supplement 1 [file tvst-13-10-37_s001.pdf]

Health Care Facility Design for Seniors and the Visually Impaired

Anne T. Riddering, OTR, COMS, CLVT
Lori Schlegel, OTR, CLVT
HFHS Center for Vision Rehabilitation and Research

1

SiteWise Project

- Enhance the safe participation of seniors in their communities, institutions and businesses

By:
- Raising awareness of accessibility issues
- Promoting easy, inexpensive adaptations
- Educating decision-makers, staff

2

The Good News: We're Living Longer

Life Expectancies (Caucasians)

	1900	1950	2000
Men	46 yrs	66 yrs	74 yrs
Women	48 yrs	71 yrs	80 yrs

3

The Bad News: The Cost of Longevity

30% > age 65 have 3 or more Chronic Diseases

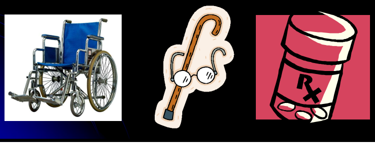

4

Epidemic of Vision Loss in Seniors

There are more
visually impaired <u>older than 65</u>
than everyone under 65

5

#1 Cause Of Vision Loss Age-Related Macular Degeneration

- > 13 million now
- More than double in 20 yrs

6

Macular Degeneration

	# Seniors	#/yr Severely visually impaired
2005	38 Million	200,000
2030	88 Million	500,000

*Javitt J. et al. Ophthal. 2003.

7

The Other Bad News: Macular Degeneration is not going away anytime soon

1. Age
2. Predisposition (Caucasian)
3. Environmental Exposures
4. Food Supply

8

Macular Degeneration:

Gradual loss of central, detailed vision

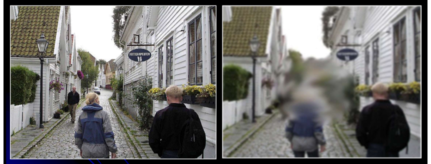

9

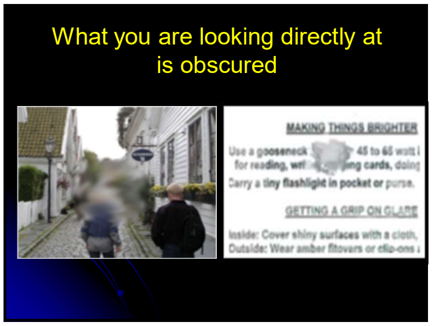

10

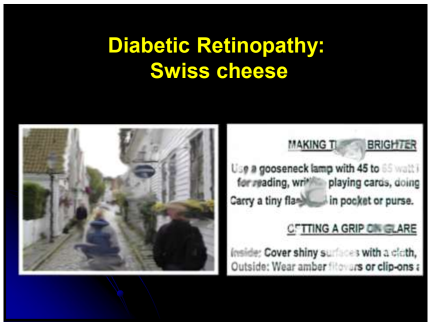

11

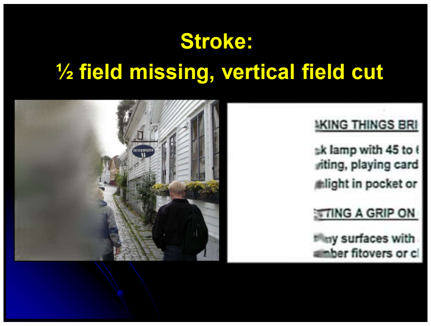

12

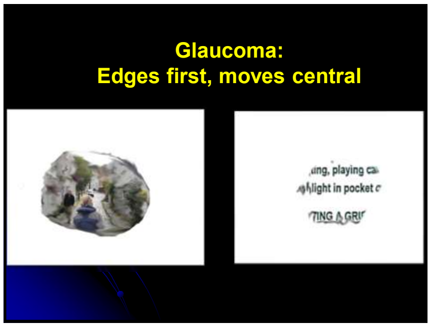

13

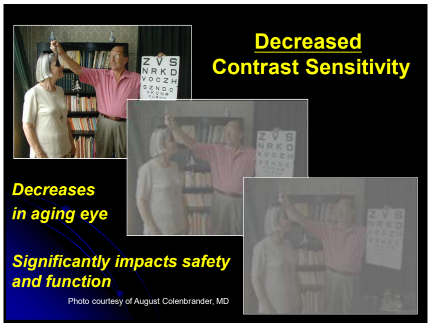

14

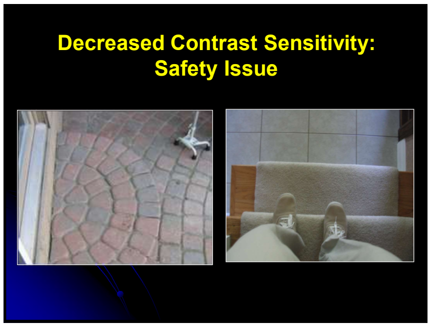

15

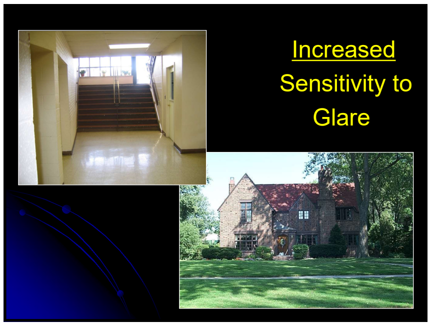

16

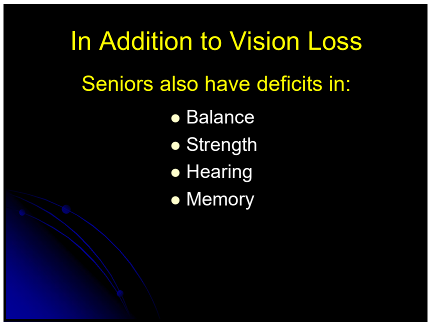

17

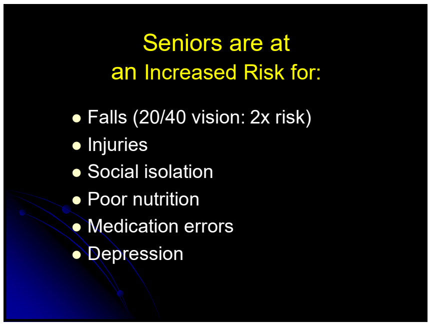

18

Central Vision Loss:
INVISIBLE to observers

19

Invisible in Law

Full Sight ------ - - - ----- Blindness
No problem **ADA**

Low Vision
FAR more prevalent than blindness
FAR more likely to be injured

20

HFHS
Center for Vision and Neuro-Rehabilitation & Research

Individual rehabilitation to maximize independence, safety and wellbeing

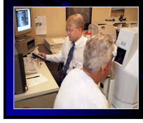
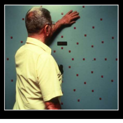
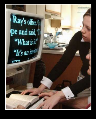

21

In the Community

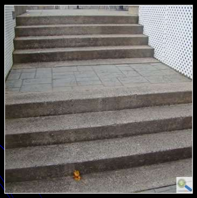

22

SiteWise
Super Suggestions for:

- **Medical Centers**
- **Pharmacies**
- Banks
- Restaurants
- Libraries
- Community Centers
- Places of Worship
- Public Spaces

23

Senior-Friendly Health Care Facilities:
The Issues

- **Physical:** Risk for trips, falls, injuries
- **Functional:** Inabilitiy to
  - Read signs, directions, instructions
  - Complete forms, financial transactions
  - Navigate safely

24

Senior-Friendly Health Care Facilities:
The Issues

- **Psychological:**
  - Fear of falling
  - Appearance of Incompetence
  - Instant young-old to old-old
  - Embarrassment
  - Depression
  - Already vulnerable in medical setting

25

Senior Trends

- More Educated:

	High School	College
1970:	28%	
1998:	67%	
2010		15%
2030:	83%	24%

- More Demanding Consumers

26

Impact on Healthcare institution of being INaccessible to Seniors

- Liability from injuries
- Decreased customer satisfaction
- Loss of customer loyalty, referrals
- Decreased income

27

Impact on Health Care Institution of being ACCESSIBLE to Seniors

- Great public relations
- Great customer relations
- Great public service
- Increased satisfaction and loyalty among seniors, their families and friends
- Increased income

Good Medicine · Good Business

28

The SiteWise Goal

- Make easy, inexpensive adaptations to existing structures, inside/outside
- Include senior-sight-friendly principles in updates, remodels, new construction and leases

29

Assessing Your Facility and Making Simple Modifications

30

SiteWise Checklist

- Simple Yes/No format
- Aimed to assist in identifying areas in facility that impact
  - Safety
  - Accessibility
  - Service Delivery
- Simple suggestions for adaptations are available upon request

31

Maximize light, Minimize glare

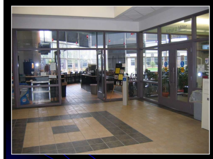
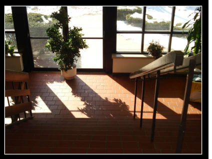

- Impacts Safety
- Impacts Accessibility

32

Maximize Contrast

- Impacts Safety
- Impacts Accessibility

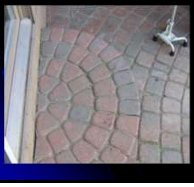
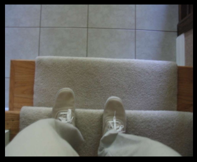

33

Increase Size

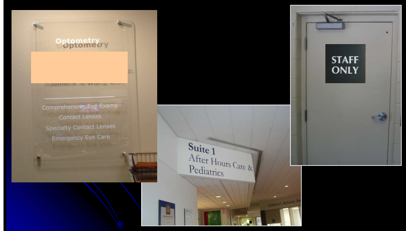

34

Remove Hazards

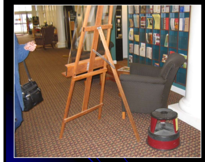
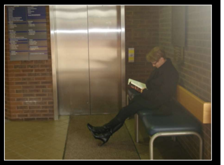

35

Parking Lots and Sidewalks

	Parking Lots/Sidewalks	Yes	No	Not Applicable
1	Are there painted crosswalks from the patient parking lot to building entrances?			
2	Are the concrete parking barriers in the parking lot painted?			
3	Are curbs on parking lot islands painted?			
4	Is uneven or broken concrete in parking lot/sidewalks identified for customer safety? (painted, orange cone)			
5	Are the top and side edges of all curbs painted?			
6	Are the inclining/declining surfaces (ramps, ramp edges) leading to all building entrances marked with paint?			
7	Are handicap curb cuts marked with paint?			
8	Are the patient drop off areas/ no parking areas painted at all building entrances?			
9	Do the painted handicap parking spaces have wheelchair accessibility on both right and left sides of vehicles?			
10	Are walkways clear of any freestanding objects (i.e. easels, plants, floor signs, displays, wheelchairs, carts)?			
11	Are all freestanding or protruding objects high contrast or marked with high contrast for customer safety?			
	Totals			

36

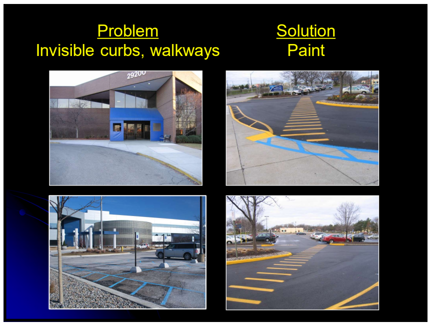

37

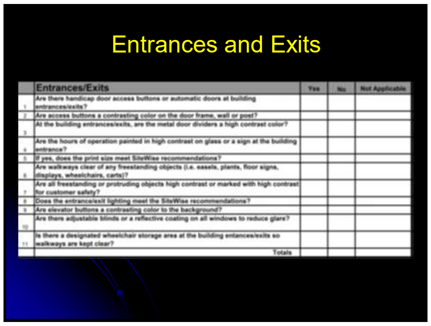

	Entrances/Exits	Yes	No	Not Applicable
1	Are there handicap door access buttons or automatic doors at building entrances/exits?			
2	Are access buttons a contrasting color on the door frame, wall or post?			
3	At the building entrances/exits, are the metal door dividers a high contrast color?			
4	Are the hours of operation painted in high contrast on glass or a sign at the building entrance?			
5	If yes, does the print size meet SiteWise recommendations?			
6	Are walkways clear of any freestanding objects (i.e. easels, plants, floor signs, displays, wheelchairs, carts)?			
7	Are all freestanding or protruding objects high contrast or marked with high contrast for customer safety?			
8	Does the entrance/exit lighting meet the SiteWise recommendations?			
9	Are elevator buttons a contrasting color to the background?			
10	Are there adjustable blinds or a reflective coating on all windows to reduce glare?			
11	Is there a designated wheelchair storage area at the building entances/exits so walkways are kept clear?			
	Totals			

38

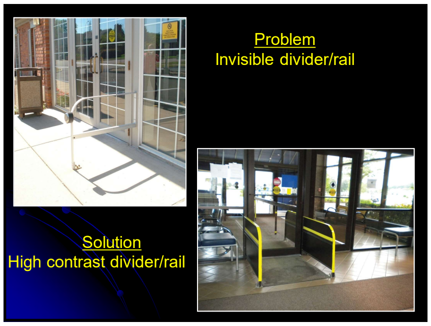

39

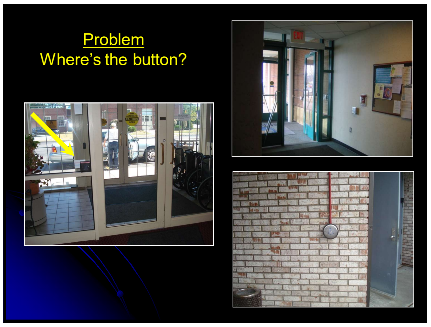

40

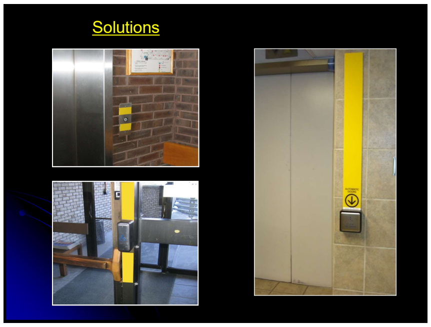

41

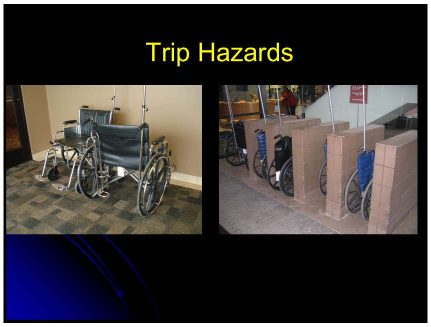

42

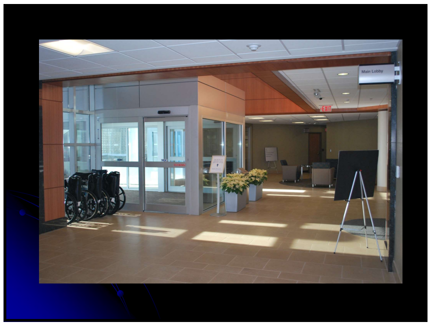

43

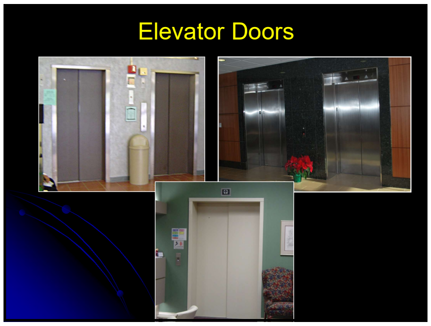

44

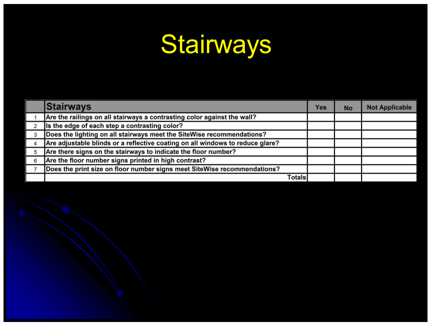

	Stairways	Yes	No	Not Applicable
1	Are the railings on all stairways a contrasting color against the wall?			
2	Is the edge of each step a contrasting color?			
3	Does the lighting on all stairways meet the SiteWise recommendations?			
4	Are adjustable blinds or a reflective coating on all windows to reduce glare?			
5	Are there signs on the stairways to indicate the floor number?			
6	Are the floor number signs printed in high contrast?			
7	Does the print size on floor number signs meet SiteWise recommendations?			
	Totals			

45

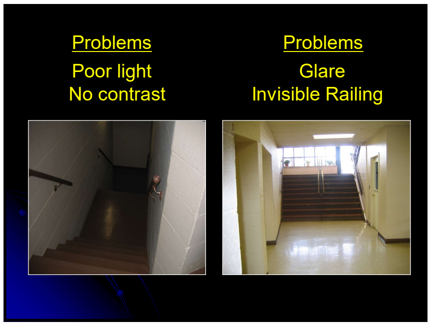

46

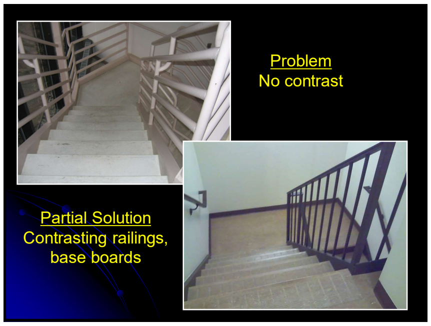

47

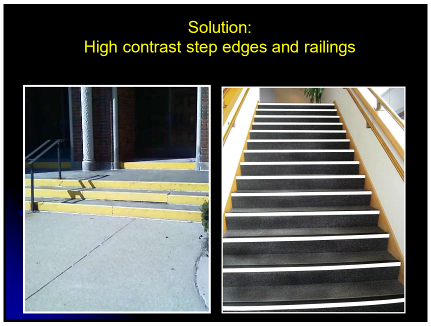

48

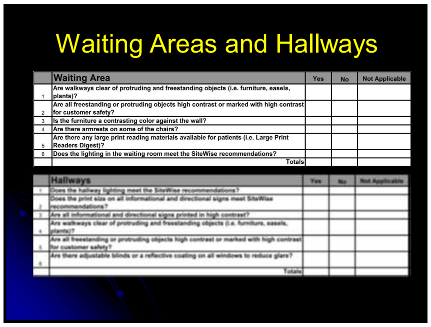

	Waiting Area	Yes	No	Not Applicable
1	Are walkways clear of protruding and freestanding objects (i.e. furniture, easels, plants)?			
2	Are all freestanding or protruding objects high contrast or marked with high contrast for customer safety?			
3	Is the furniture a contrasting color against the wall?			
4	Are there armrests on some of the chairs?			
5	Are there any large print reading materials available for patients (i.e. Large Print Readers Digest)?			
6	Does the lighting in the waiting room meet the SiteWise recommendations?			
	Totals			

	Hallways	Yes	No	Not Applicable
1	Does the hallway lighting meet the SiteWise recommendations?			
2	Does the print size on all informational and directional signs meet SiteWise recommendations?			
3	Are all informational and directional signs printed in high contrast?			
4	Are walkways clear of protruding and freestanding objects (i.e. furniture, easels, plants)?			
5	Are all freestanding or protruding objects high contrast or marked with high contrast for customer safety?			
6	Are there adjustable blinds or a reflective coating on all windows to reduce glare?			
	Totals			

49

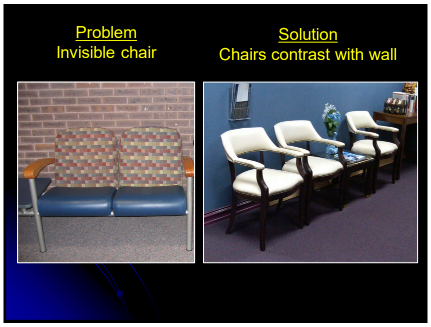

50

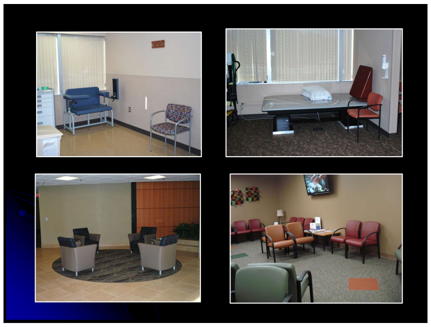

51

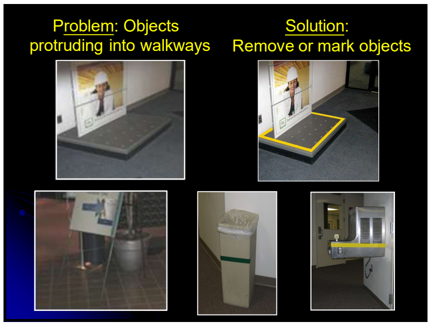

52

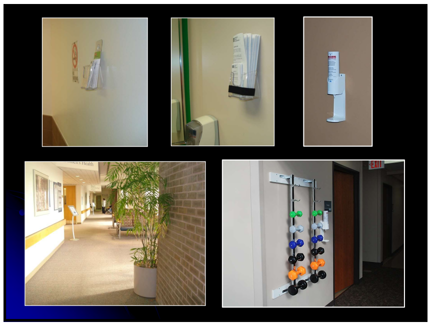

53

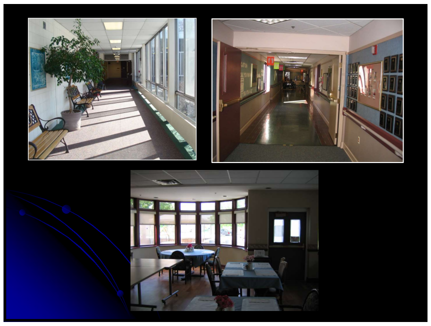

54

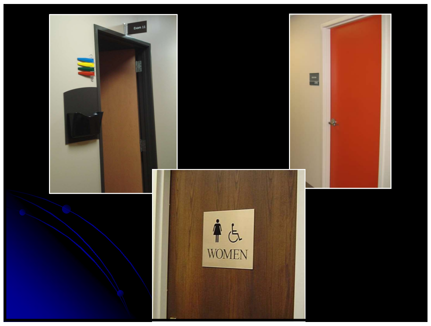

55

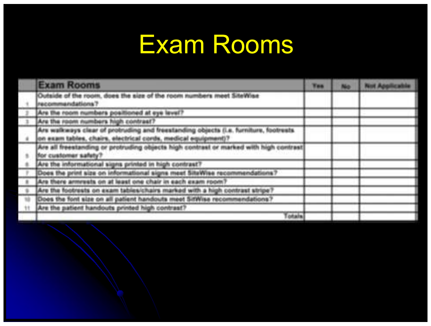

	Exam Rooms	Yes	No	Not Applicable
1	Outside of the room, does the size of the room numbers meet SiteWise recommendations?			
2	Are the room numbers positioned at eye level?			
3	Are the room numbers high contrast?			
4	Are walkways clear of protruding and freestanding objects (i.e. furniture, footrests on exam tables, chairs, electrical cords, medical equipment)?			
5	Are all freestanding or protruding objects high contrast or marked with high contrast for customer safety?			
6	Are the informational signs printed in high contrast?			
7	Does the print size on informational signs meet SiteWise recommendations?			
8	Are there armrests on at least one chair in each exam room?			
9	Are the footrests on exam tables/chairs marked with a high contrast stripe?			
10	Does the font size on all patient handouts meet SitWise recommendations?			
11	Are the patient handouts printed high contrast?			
	Totals			

56

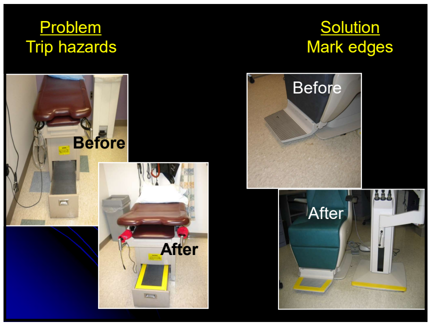

57

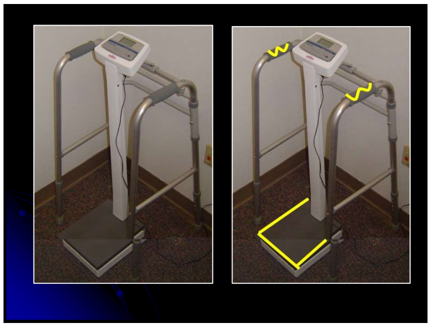

58

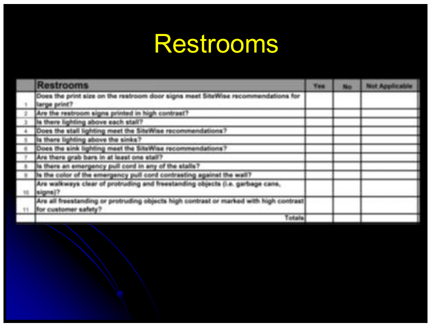

	Restrooms	Yes	No	Not Applicable
1	Does the print size on the restroom door signs meet SiteWise recommendations for large print?			
2	Are the restroom signs printed in high contrast?			
3	Is there lighting above each stall?			
4	Does the stall lighting meet the SiteWise recommendations?			
5	Is there lighting above the sinks?			
6	Does the sink lighting meet the SiteWise recommendations?			
7	Are there grab bars in at least one stall?			
8	Is there an emergency pull cord in any of the stalls?			
9	Is the color of the emergency pull cord contrasting against the wall?			
10	Are walkways clear of protruding and freestanding objects (i.e. garbage cans, signs)?			
11	Are all freestanding or protruding objects high contrast or marked with high contrast for customer safety?			
	Totals			

59

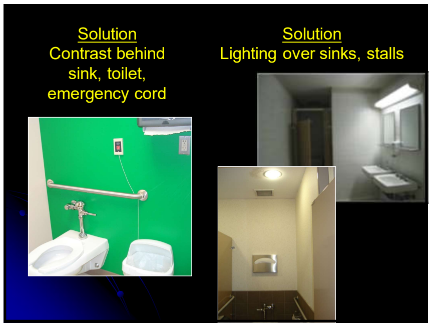

60

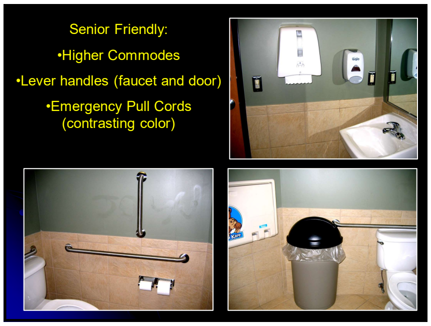

61

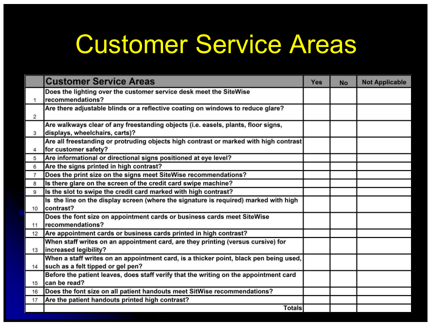

	Customer Service Areas	Yes	No	Not Applicable
1	Does the lighting over the customer service desk meet the SiteWise recommendations?			
2	Are there adjustable blinds or a reflective coating on windows to reduce glare?			
3	Are walkways clear of any freestanding objects (i.e. easels, plants, floor signs, displays, wheelchairs, carts)?			
4	Are all freestanding or protruding objects high contrast or marked with high contrast for customer safety?			
5	Are informational or directional signs positioned at eye level?			
6	Are the signs printed in high contrast?			
7	Does the print size on the signs meet SiteWise recommendations?			
8	Is there glare on the screen of the credit card swipe machine?			
9	Is the slot to swipe the credit card marked with high contrast?			
10	Is the line on the display screen (where the signature is required) marked with high contrast?			
11	Does the font size on appointment cards or business cards meet SiteWise recommendations?			
12	Are appointment cards or business cards printed in high contrast?			
13	When staff writes on an appointment card, are they printing (versus cursive) for increased legibility?			
14	When a staff writes on an appointment card, is a thicker point, black pen being used, such as a felt tipped or gel pen?			
15	Before the patient leaves, does staff verify that the writing on the appointment card can be read?			
16	Does the font size on all patient handouts meet SitWise recommendations?			
17	Are the patient handouts printed high contrast?			
	Totals			

62

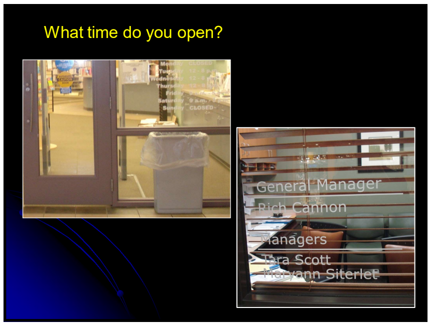

63

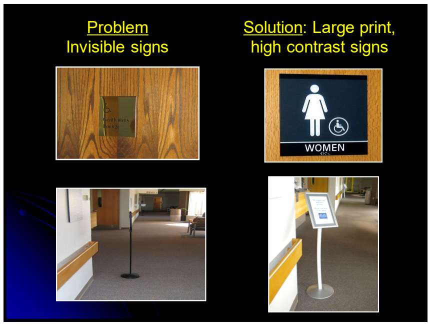

64

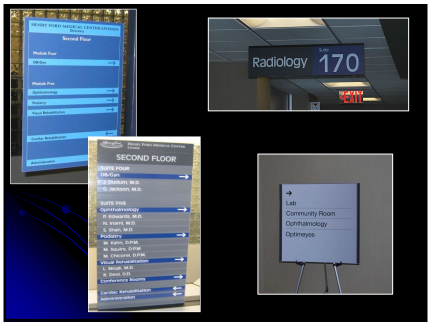

65

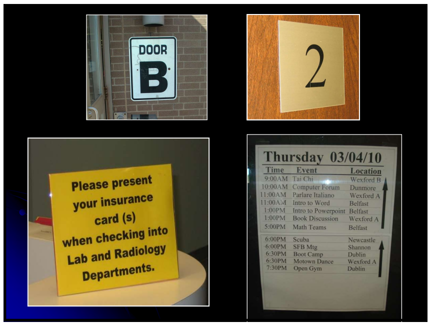

66

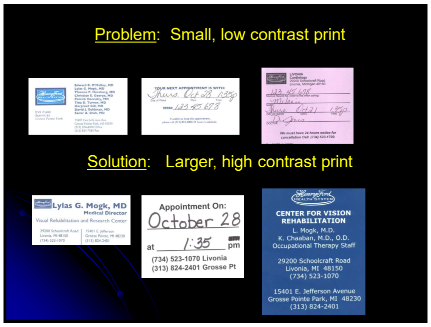

67

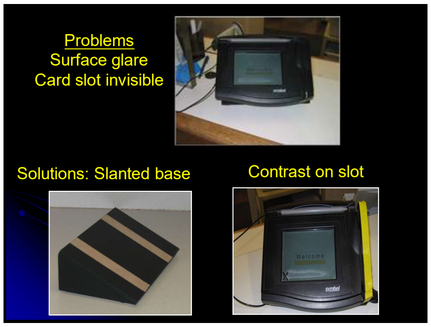

68

SiteWise Roll-out in Henry Ford Health System

- Review team evaluated 41 sites
  - 5 hospitals
    - 4: 200-400 beds; 1 80-bed psych
    - Detroit Main Campus not included in preliminary results
  - 35 outpatient offices and medical centers

69

Preliminary Results

- Gold Standard: 90-100%
- Silver: 70-90%
  1 Hospitals,12 Outpatient facilities
- Bronze: 60-70%
  1 Hospital, 7 Outpatient facilities

70

Summary

- Average Score: 62.28%
- Lowest scores
  - parking lots
  - entrances/exits
  - exam rooms
- Highest scores
  - waiting rooms
  - rest rooms

71

SiteWise
Senior-Friendly Health Facilities

- Increased safety for seniors
- Visually friendly environment for everyone
- Increased awareness of accessibility issues
- Pro-active, knowledgeable staff

72

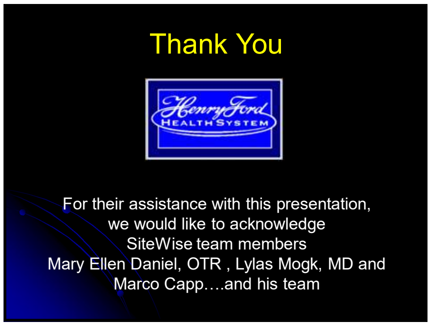
Thank You
Henry Ford
HEALTH SYSTEM
For their assistance with this presentation,
we would like to acknowledge
SiteWise team members
Mary Ellen Daniel, OTR , Lylas Mogk, MD and
Marco Capp….and his team

73
