# Supplementary material for: Assessing Eye Clinic Accessibility: A Study Validating and Applying the SiteWise Survey
Source: Transl Vis Sci Technol. 2024 Oct 29;13(10):37. doi: 10.1167/tvst.13.10.37 (PMC11534020; doi:10.1167/tvst.13.10.37)
Supplement: Supplement 2 [file tvst-13-10-37_s002.pdf]

SiteWise™

Building Name/Location ________________________

Henry Ford HEALTH SYSTEM

Please check one response for each question. If question does not apply to the building or room (e.g. no stairs, no elevator, or no windows) , check "Not Applicable". If you do not have a light meter, check "Not Applicable."

Additional pages can be added as needed for duplicate rooms (e.g. two restrooms in different locations within facility). Eliminate page(s) if not required for building being assessed (e.g. exam rooms can be eliminated for non-medical buildings).

	Parking Lots/Sidewalks	Yes	No	Not Applicable
1	Are there painted crosswalks from the patient parking lot to building entrances?			
2	Are the concrete parking barriers in the parking lot painted?			
3	Are the parking lot island curbs painted?			
4	Is uneven or broken concrete in parking lot/sidewalks identified for customer safety? (painted, orange cone)			
5	Are the edges of all curbs painted?			
6	Are the inclining/declining surfaces (ramps, ramp edges) leading to all building entrances marked with paint?			
7	Are handicap curb cuts marked with paint?			
8	Are the patient drop off areas/ no parking areas painted at all building entrances?			
9	Do handicap parking spaces have wheelchair accessibility on both right and left sides of vehicles?			
10	Are walkways clear of any freestanding objects (i.e. plantars, trash containers,valet signs, benches, carts, wheelchairs, etc.)?			
11	Are objects that cannot be removed high contrast or marked with high contrast? (e.g. permanently fixed objects or something too heavy to be easily moved such as a concrete barrier, sign or light pole, bench, or trash receptable)			
	Subtotals			

© 2010 Henry Ford Health System
Center for Vision Rehabilitation Research

SiteWise™

Building Name/Location ____________________

Henry Ford Health System

	Entrances/Exits	Yes	No	Not Applicable
1	Are there automatic doors at building entrances/exits? (e.g. automatic sliding or activated by an access button)			
2	If handicap access buttons are available, do they contrast with the background?			
3	Are the metal door dividers a high contrast color?			
4	Are the hours of operation posted at the building entrance?			
5	Is the hours of operation sign printed in high contrast?			
6	Does the print size of the hours of operation sign meet SiteWise recommendations for signs? (Approx. 1")			
7	Are walkways clear of any freestanding objects (i.e. easels, plants, floor signs, displays, wheelchairs, carts, newspaper stands, etc)?			
8	Are objects that cannot be removed high contrast or marked with high contrast? (e.g. permanently fixed objects or something too heavy to be easily moved such as a sign or pole, large planter box, bench, concrete or large trash container)			
9	Does the entrance/exit lighting meet the SiteWise recommendations? (600-800 lux)			
10	Are elevator buttons a contrasting color to the background?			
11	Are there adjustable blinds or a reflective coating on all windows to reduce glare?			
12	Is there a designated storage area for wheelchairs to keep walkways clear?			
	Subtotals			

© 2010 Henry Ford Health System
Center for Vision Rehabilitation Research

SiteWise™

Building Name/Location ____________________

Henry Ford Health System

	Hallways	Yes	No	Not Applicable
1	Does the hallway lighting meet the SiteWise recommendations? (400-600 lux)			
2	Does the print size on all informational and directional signs meet SiteWise recommendations? (Approx. 1")			
3	Are all informational and directional signs printed in high contrast?			
4	Are walkways clear of protruding and freestanding objects (i.e. furniture, easels, plants, trash containers, signs, etc.)?			
5	Are objects that cannot be removed high contrast or marked with high contrast? (e.g. permanently fixed objects or something too heavy to be easily moved such as a large potted plant or tree, bench or fixed furniture, piano, drinking fountain, hand sanitizer dispenser, self check-in kiosk, ATM machine, or pillar)			
6	Are there adjustable blinds or a reflective coating on all windows to reduce glare?			
	Subtotals			

© 2010 Henry Ford Health System
Center for Vision Rehabilitation Research

SiteWise™

Building Name/Location ____________________________

Henry Ford Health System

	Stairways	Yes	No	Not Applicable
1	Are the railings on all stairways a contrasting color against the wall?			
2	Is the edge of each step a contrasting color?			
3	Does the lighting on all stairways meet the SiteWise recommendations? (400-600 lux)			
4	Are adjustable blinds or a reflective coating on all windows to reduce glare?			
5	Are there signs on the stairways to indicate the floor number?			
6	Are the floor number signs printed in high contrast?			
7	Does the print size on floor number signs meet SiteWise recommendations? (Approx. 6")			
	Subtotals			

© 2010 Henry Ford Health System
Center for Vision Rehabilitation Research

SiteWise™

Building Name/Location ___________________________

Henry Ford Health System

	Waiting Areas	Yes	No	Not Applicable
1	Are walkways clear of protruding and freestanding objects (i.e. furniture, easels, plants)?			
2	Are objects that cannot be removed high contrast or marked with high contrast? (e.g. permanently fixed objects or something too heavy to be easily moved such as a large potted plant or tree, pillar, hand stanitizer dispenser or station, or self check-in kiosk)			
3	Is the furniture a contrasting color against the wall?			
4	Are there armrests on some of the chairs?			
5	Are there any large print reading materials available for patients (i.e. Large Print Readers Digest)?			
6	Does the lighting in the waiting room meet the SiteWise recommendations? (400-600 lux) (*note: an internal ophthalmology waiting room (a dilating room) may be more dimly lit)			
7	Are adjustable blinds or a reflective coating on all windows to reduce glare?			
	Subtotals			

© 2010 Henry Ford Health System
Center for Vision Rehabilitation Research

SiteWise™

Building Name/Location ____________________________

Henry Ford HEALTH SYSTEM

	Customer Service Areas	Yes	No	Not Applicable
1	Does the lighting over the customer service desk meet the SiteWise recommendations? (600-800 lux)			
2	Are there adjustable blinds or a reflective coating on windows to reduce glare?			
3	Are walkways clear of any freestanding objects (i.e. easels, plants, floor signs, displays, wheelchairs, carts)?			
4	Are objects that cannot be removed high contrast or marked with high contrast? (e.g. permanently fixed objects or something too heavy to be easily moved such as a pillar, large potted plant or tree, furniture or desk, or trash container)			
5	Are informational or directional signs positioned at eye level? (any part of the sign is 50-60" from the floor)			
6	Are the signs printed in high contrast?			
7	Does the print size on the signs meet SiteWise recommendations? (approx. 1")			
8	Is glare elimintated from the screen of the credit card swipe machine? (If the machine tilts, it should effectively eliminate glare.)			
9	Is the slot to swipe the credit card marked with high contrast?			
10	Is the line on the display screen (where the signature is required) marked with high contrast?			
11	Does the font size on appointment or business cards meet SiteWise recommendations? (key information 16 point bold font-Arial, Tahoma, Verdana)			
12	Is significant information on appointment cards or business cards (e.g. name, phone number, date, time) printed in high contrast?			
13	Does staff print (versus cursive writing) on appointment cards for increased readability?			
14	Is a thicker point, black pen, such as a felt tipped or gel pen, being used when a staff writes on appointment cards?			
15	Before the customer leaves, does staff verify that the writing on the appointment card can be read?			
16	Does the font size on all customer handouts meet SitWise recommendations? (12-16 point bold font-Arial, Tahoma, Verdana)			
17	Are the customer educational/informational handouts printed high contrast?			
	© 2010 Henry Ford Health System Center for Vision Rehabilitation Research — Subtotals			

SiteWise™

Building Name/Location ___________________________

Henry Ford Health System

	Restrooms	Yes	No	Not Applicable
1	Is the restroom for use by one person at a time (i.e. no stalls)?			
2	Does the print size on the restroom door signs meet SiteWise recommendations for large print? (Approx. 6" symbols)			
3	Are the restroom signs printed in high contrast?			
4	Is there lighting above each stall? (If there is no stall, check Not Applicable)			
5	Does the stall lighting meet the SiteWise recommendations? (400-600 lux)			
6	Is there lighting above the sinks?			
7	Does the sink lighting meet the SiteWise recommendations? (400-600 lux)			
8	Are there grab bars near at least one commode?			
9	Is there an emergency pull cord near at least one commode?			
10	Is the color of the emergency pull cord contrasting against the wall?			
11	Are walkways clear of protruding and freestanding objects (i.e. garbage cans, signs)?			
12	Are objects that cannot be removed high contrast or marked with high contrast? (e.g. permanently fixed objects or something too heavy to be easily moved such as a trash receptacle, or towel dispenser)			
	Subtotals			

© 2010 Henry Ford Health System
Center for Vision Rehabilitation Research

SiteWise™

Building Name/Location ____________________________

Henry Ford Health System

	Exam Rooms	Yes	No	Not Applicable
1	Does the size of the room number meet SiteWise recommendations? (Approx. 3")			
2	Are the room numbers positioned at eye level? (any part of the sign is 50-60" from the floor)			
3	Are the room numbers high contrast?			
4	Are walkways clear of protruding and freestanding objects (i.e. furniture, footrests on exam tables, chairs, electrical cords, medical equipment, scales)?			
5	Are objects that cannot be removed high contrast or marked with high contrast? (e.g. permanently fixed objects or something too heavy to be easily moved such as an exam table or chair or a desk)			
6	Are the informational signs printed in high contrast?			
7	Does the print size on informational signs meet SiteWise recommendations? (Approx. 1")			
8	Are there armrests on at least one chair in each exam room?			
9	Are the footrests on exam tables/chairs marked with a high contrast stripe?			
10	Does the font size on all patient handouts meet SitWise recommendations? (12-16 point bold font-Arial, Tahoma, Verdana)			
11	Are the patient handouts printed high contrast?			
	Subtotals			

© 2010 Henry Ford Health System
Center for Vision Rehabilitation Research

SiteWise™

Building Name/Location ________________________

Henry Ford Health System

	Room ______________________________ (This sheet can be copied and used for additonal rooms.)	Yes	No	Not Applicable
1	Does the print size of the room name and number meet SiteWise recommendations? (Approx. 2 for office or suite numbers)			
2	Is the room name and number positioned at eye level? (any part of the sign is 50-60" from the floor)			
3	Is the room name and number printed in high contrast?			
4	Does the lighting meet the SiteWise recommendations? (400-600 lux)			
5	Are the informational signs printed in high contrast?			
6	Does the print size on informational signs meet SiteWise recommendations? (Approx. 1")			
7	Do some of the chairs have armrests?			
8	Are there any freestanding or protruding objects in the walkway? (i.e. furniture, plants, floor signs, easels)			
9	Are objects that cannot be removed high contrast or marked with high contrast? (e.g. permanently fixed objects or something too heavy to be easily moved such as a sign or support pillar, fixed bench or furniture, or conference room table)			
10	Are adjustable blinds or a reflective coating on all windows to reduce glare?			
	Subtotals			

© 2010 Henry Ford Health System
Center for Vision Rehabilitation Research

SiteWise™

Building Name/Location ________________________

Henry Ford Health System

Scoring For the SiteWIse Checklist

Instructions:

Step 1: After totaling the responses on each checklist page, copy the totals to this page.

**Subtotals (from previous pages)**	**Yes**	**No**	**Not Applicable**
Page 1: Parking Lots and Sidewalks			
Page 2: Entrances and Exits			
Page 3: Hallways			
Page 4: Stairways			
Page 5: Waiting Areas			
Page 6: Customer Service Areas			
Page 7: Restrooms			
Page 8: Exam Rooms			
Additional Page ___:			
Additional Page ___:			
Additional Page ___:			
Additional Page ___:			
Additional Page ___:			
Additional Page ___:			
**Step 2:** Add up subtotals to get **TOTAL**			

Step 3: Add together the number of "Yes" and "No" responses.............. __________

Step 4: Divide and calculate a percentage score. Use lines below for calculation.

A. Number of "Yes" responses (Step 2 Total) ÷ from Step 3 number (comination Yes & No).

B. Multiply by 100 to calculate percentage.

Yes _______ ÷ Step 3 Number _________ = ___________X 100 = ________%

Key For SiteWise Standard

90% and higher	Outstanding	(Gold Standard)
71-89 %	Adequate	(Silver Standard)
60-70%	Minimum	(Bronze Standard)

© 2010 Henry Ford Health System
Center for Vision Rehabilitation Research
